# Supplementary material for: Dynamic transcriptomic profiles of zebrafish gills in response to zinc supplementation
Source: BMC Genomics. 2010 Oct 11;11:553. doi: 10.1186/1471-2164-11-553 (PMC3091702; doi:10.1186/1471-2164-11-553)
Supplement: Additional file 2 — Interactive Direct Interaction Network representing the molecular interactions between zinc, copper, iron, calcium and proteins encoded by transcripts changed by zinc supplementation. Mini web-site containing index.html and hyperlinked pages in subdirectory describing a Direct Interaction Network automatically generated based on curated interactions contained within the proprietary PathwayArchitect database. Ovals represent proteins and the circles symbolize metal ions. Objects are coloured by their abundance in zebrafish at the time-point they were significantly different from the control is a scale from -4 fold (dark green) to +4 fold (dark red). Where significant differences were found at more than one time-point, the colour overlay shows expression at the first instance. Dark blue squares denote 'binding', and light blue squares 'expression'; green squares stand for 'regulation', green diamonds for 'metabolism', and green circles for 'promoter binding'. Arrow heads indicate directionality of the interaction where annotated. All nodes and edges can be further interrogated by selecting the relative area of the image. [file 1471-2164-11-553-S2.zip › PathwayArchitect Zn xs DIN/143870.html]

# PROTEIN: S100A1

|  |  |
| --- | --- |
| Name | S100A1 |
| Type | PROTEIN |
| Description | S100 calcium binding protein A1 |
| Note | The protein encoded by this gene is a member of the S100 family of proteins containing 2 EF-hand calcium-binding motifs. S100 proteins are localized in the cytoplasm and/or nucleus of a wide range of cells, and involved in the regulation of a number of cellular processes such as cell cycle progression and differentiation. S100 genes include at least 13 members which are located as a cluster on chromosome 1q21. This protein may function in stimulation of Ca2+-induced Ca2+ release, inhibition of microtubule assembly, and inhibition of protein kinase C-mediated phosphorylation. Reduced expression of this protein has been implicated in cardiomyopathies. |
| Alias | S100a |
|  | S100A1 protein |
|  | S100A |
|  | S-100 protein, alpha chain |
|  | S100A1 |
|  | S100a1 |
|  | S100 |
|  | AI266795 |
|  | S100 alpha |
|  | S100 protein, alpha polypeptide |
|  | S100 calcium-binding protein A1 |


---

|  |  |
| --- | --- |
| GO Component | sarcoplasmic reticulum |
|  | protein complex |


---

|  |  |
| --- | --- |
| GO ID | GO:0051117 |
|  | GO:0042803 |
|  | GO:0016529 |
|  | GO:0000122 |
|  | GO:0046872 |
|  | GO:0043234 |
|  | GO:0005509 |
|  | GO:0008016 |
|  | GO:0007275 |
|  | GO:0007242 |


---

|  |  |
| --- | --- |
| MIM | MIM:176940 |


---

|  |  |
| --- | --- |
| Connectivity | 180 |


---

|  |  |
| --- | --- |
| Entrez ID | 295214 |
|  | 20193 |
|  | 6271 |


---

|  |  |
| --- | --- |
| Agilent ID | A\_53\_P148979 |
|  | A\_44\_P259957 |
|  | A\_51\_P172475 |
|  | A\_43\_P15752 |
|  | A\_23\_P383227 |


---

|  |  |
| --- | --- |
| Cellular Localization | Endoplasmic reticulum |
|  | Cytoplasm |
|  | Organelle |
|  | Cell |


---

|  |  |
| --- | --- |
| Pathway | Zn xs inventory |
|  | Zn xs DIN |


---

|  |  |
| --- | --- |
| GO Process | negative regulation of transcription from RNA polymerase II promoter |
|  | intracellular signaling cascade |
|  | development |
|  | regulation of heart contraction rate |


---

|  |  |
| --- | --- |
| UniGene | Mm.24662 |
|  | Hs.515715 |
|  | Rn.11091 |


---

|  |  |
| --- | --- |
| Affymetrix Probeset ID | 1388456\_at |
|  | 1417421\_at |
|  | 1419814\_s\_at |
|  | 205334\_at |
|  | 34674\_at |
|  | 95452\_i\_at |
|  | 95453\_f\_at |
|  | 95454\_at |
|  | g5454031\_3p\_at |
|  | g5454031\_3p\_x\_at |
|  | rc\_AI228548\_at |
|  | rc\_AI228548\_g\_at |
|  | S68809\_s\_at |
|  | U26356mRNA\_s\_at |
|  | w08053\_at |
|  | X58079\_at |
|  | TC39623\_i\_at |
|  | TC39623\_s\_at |


---

|  |  |
| --- | --- |
| GO Function | ATPase binding |
|  | protein homodimerization activity |
|  | metal ion binding |
|  | calcium ion binding |


---

|  |  |
| --- | --- |
| Nucleotide | AK002721 |
|  | AF218353 |
|  | AF368423 |
|  | AL162258 |
|  | AA500563 |
|  | X58079 |
|  | AK017279 |
|  | BC014392 |
|  | S68809 |
|  | AA207749 |
|  | M65210 |
|  | AF087687 |
|  | U26358 |
|  | AA000715 |
|  | AK126904 |
|  | AK012578 |
|  | BC005590 |
|  | NM\_011309 |
|  | NM\_001007636 |
|  | BT006938 |
|  | BC035791 |
|  | AK168822 |
|  | AA432539 |
|  | NM\_006271 |


---

|  |  |
| --- | --- |
| Protein | AAF32320 |
|  | AAC64108 |
|  | AAL14436 |
|  | AAB53657 |
|  | CAI19674 |
|  | NP\_006262 |
|  | CAI19675 |
|  | NP\_001007637 |
|  | P35467 |
|  | NP\_035439 |
|  | BAE40649 |
|  | AAH14392 |
|  | P56565 |
|  | AAH05590 |
|  | CAI19676 |
|  | AAB20539 |
|  | BAB28330 |
|  | CAA41107 |
|  | BAB30670 |
|  | P23297 |
|  | BAB22308 |
|  | AAP35584 |
|  | CAI19677 |


---

|  |  |
| --- | --- |
| Organism | Mammal |


---

|  |  |
| --- | --- |
| Location | chromosome 3, 3 43.6 cM, 3 F1-F2 (Mus musculus) |
|  | chromosome 2, 2q34 (Rattus norvegicus) |
|  | chromosome 1, 1q21 (Homo sapiens) |
|  | 3 43.6 cM (Mus musculus) |


---

|  |  |
| --- | --- |
